# Membranotropic Cell Penetrating Peptides: The Outstanding Journey

**DOI:** 10.3390/ijms161025323

**Published:** 2015-10-23

**Authors:** Annarita Falanga, Massimiliano Galdiero, Stefania Galdiero

**Affiliations:** 1 Department of Pharmacy, University of Naples “Federico II”, Via Mezzocannone 16, 80134 Naples, Italy; E-Mail: annarita.falanga@unina.it; 2 CiRPEB, University of Naples “Federico II”, Via Mezzocannone 16, 80134 Naples, Italy; E-Mail: massimiliano.galdiero@unina2.it; 3 Department of Experimental Medicine, II University of Naples, Via De Crecchio 7, 80138 Naples, Italy

**Keywords:** membranotropic peptides, hydrophobicity, fusion, delivery

## Abstract

The membrane bilayer delimits the interior of individual cells and provides them with the ability to survive and function properly. However, the crossing of cellular membranes constitutes the principal impediment to gaining entry into cells, and the potential therapeutic application of many drugs is predominantly dependent on the development of delivery tools that should take the drug to target cells selectively and efficiently with only minimal toxicity. Cell-penetrating peptides are short and basic peptides are widely used due to their ability to deliver a cargo across the membrane both *in vitro* and *in vivo*. It is widely accepted that their uptake mechanism involves mainly the endocytic pathway, the drug is catched inside endosomes and lysosomes, and only a small quantity is able to reach the intracellular target. In this wide-ranging scenario, a fascinating novel hypothesis is that membranotropic peptides that efficiently cross biological membranes, promote lipid-membrane reorganizing processes and cause a local and temporary destabilization and reorganization of the membrane bilayer, may also be able to enter cells circumventing the endosomal entrapment; in particular, by either favoring the escape from the endosome or by direct translocation. This review summarizes current data on membranotropic peptides for drug delivery.

## 1. Introduction

The plasma membrane is a highly effective and selective barrier that determines the ability of cells to survive and function properly. However, this barrier also constitutes a primary obstacle for intracellular delivery of theranostics. Notwithstanding their potentiality, a number of novel molecules do not reach the pharmaceutical stage and do not tickle industrial interest because of their low cell membrane permeability; in fact, these molecules should be delivered intracellularly to induce a biological effect. Thus, a key issue to enhance the therapeutic potential of a drug is the development of delivery tools which should take the drug to target cells selectively and efficiently with only minimal toxicity. The blood brain barrier (BBB), is a dynamic interface preventing transport of most drugs from the vasculature into the brain parenchyma [1] and thus delivering a drug across the BBB is an even more difficult task. Improving the drug cellular uptake represents a great challenge for scientists working in the field of drug development.

Over the past few decades, nanotechnology has been successfully exploited in design numerous platforms for theranostics, each presenting its own advantages and disadvantages. In particular, several nanocarriers (polymers, nanoparticles, liposomes, micelles, and dendrimers) have been extensively studied for drug delivery to cells [2,3,4,5,6]. Their delivery is generally dependent on passive accumulation in the pathological regions; thus, the delivery is not specific and healthy tissues are also involved. Recently, extensive literature has described the use of short cationic and/or amphipathic peptides, usually known as cell-penetrating peptides (CPPs), for mediating drug delivery due to their intrinsic ability to enter cells and mediate uptake of a wide range of macromolecular cargoes [7,8,9,10]. Thus, CPPs have huge potential in biotechnology, but, in order to achieve this potential, it will be important to understand how they cross biological membranes and localize to specific intracellular compartments. Their main features are low cytotoxicity, ability to internalize into a variety of cell types, dose-dependent efficiency, no restriction with respect to the size or type of cargo, ability to transport covalently or non-covalently conjugated cargoes.

Macromolecules are usually transported into the cell by endocytosis [11] which occurs by multiple mechanisms that essentially fall into two distinct categories: phagocytosis and pinocytosis. Phagocytosis is a complex process involving the uptake of large particles and is usually restricted to specialized mammalian cells. Pinocytosis occurs in all cell types and involves the uptake of smaller particles. It is widely accepted that the main pathway of CPPs mediated uptake is endocytosis, but direct uptake by energy independent pathways have also been reported for specific CPPs. The uptake mechanism of CPPs is not fully understood, but the process seems to vary considerably from peptide to peptide [12]. It is likely that CPPs can enter cells via multiple pathways, including direct penetration of the plasma membrane and endocytic uptake according to the nature of the peptide/cell interaction [13,14,15,16].

In order to optimize the nanosystem and produce maximum effect, and enhance intracellular behavior and efficiency of cargo delivery, it is fundamental to understand the uptake mechanism and intracellular trafficking of drug carriers. Although CPPs have been widely used to deliver cargo molecules into cells, their exact uptake mechanism is still an issue and lots of answers are still sought after. It is now evident that the cellular uptake mechanisms depend on each CPP features, the carried molecule, the cell type and the membrane lipid composition. This wide variety of possibilities results in different modes and levels of uptake.

## 2. CPP Classification

CPPs have been categorized according to several criteria. Our classification is based on their hydrophilicity and hydrophobicity and thus on their different interaction with the membrane bilayer. The first group is composed of cationic CPPs. The prototype of this class is represented by the HIV-1 protein TAT [17]. These peptides have a high content of arginine, lysine and histidine residues. The arginine guanidine head group forms hydrogen bonds with the negatively charged phosphates and sulphates on the cell membrane and might lead to internalization under physiological pH conditions. The lysine is a cationic amino acid as the arginine, but it lacks the guanidine head group, and therefore is less effective at penetrating the plasma membrane. Several studies have demonstrated that, for efficient cellular uptake, it is necessary to have at least eight positive charges [18].

The second group is composed of membranotropic and amphipathic peptides, which contain hydrophobic amino acids and present a low net charge. The amphipathicity plays a crucial role in their ability to interact with the membrane bilayer and thus in their mechanism of internalization. Amphipathic CPPs are divided into: primary amphipathic CPPs, secondary amphipathic α-helical CPPs, β-sheet amphipathic CPPs, and proline-rich amphipathic CPPs [19]. These peptides are characterized by the presence of lipophilic and hydrophilic blocks or by the presence of a lipophilic and hydrophobic face that are involved in mediating the peptide translocation across the cell membrane [20]. Viral fusion peptides belong to the group of membranotropic CPPs [21]. This class of peptides will be the object of the present review (a list of examples of peptides belonging to this class is reported in Table 1).

**Table 1:** Examples of membranotropic peptides used in drug delivery.

Name	Origin	Amino Acid Sequence	Type	Reference
Pep-1	NLS from Simian Virus 40 large antigen and reverse transcriptase of HIV	KETWWETWWTEWSQPKKKRKV	Primary amphipathic	[22,23]
pVEC	VE-cadherin	LLIILRRRRIRKQAHAHSK	Primary amphipathic	[24]
VT5	Synthetic peptide	DPKGDPKGVTVTVTVTVTGKGDPKPD	β-sheet amphipathic	[25]
C105Y	1-antitrypsin	CSIPPEVKFNKPFVYLI	-	[26]
Transportan	Galanin and mastoparan	GWTLNSAGYLLGKINLKALAALAKKIL	Primary amphipathic	[27]
TP10	Galanin and mastoparan	AGYLLGKINLKALAALAKKIL	Primary amphipathic	[27]
MPG	A hydrofobic domain from the fusion sequence of HIV gp41 and NLS of SV40 T antigen	GALFLGFLGAAGSTMGA	β-sheet amphipathic	[28]
gH625	Glycoprotein gH of HSV type I	HGLASTLTRWAHYNALIRAF	Secondary amphipathic α-helical	[29]
INF	Influenza HA2 fusion peptide	GLFEAIEGFIENGWEGMIDGWYGC	Secondary amphipathic α-helical	[30]
CADY	PPTG1 peptide	GLWRALWRLLRSLWRLLWRA	Secondary amphipathic α-helical	[31]
GALA	Synthetic peptide	WEAALAEALAEALAEHLAEALAEALEALAA	Secondary amphipathic α-helical	[32]

## 3. Membranotropic Peptides

Membranotropic peptides are a class of peptides playing a prominent role in biology. Their simultaneous hydrophobic and amphipathic nature determines their high propensity for binding to lipid membranes. To look at the valuable features possessed by membranotropic peptides in more detail, one should start with their physical properties. Membranotropic peptides are characterized by the presence of an unusually high content of alanine and glycine residues and often also prolines. This high content of Ala/Gly determines their peculiar features compared to signal sequences and transmembrane anchors; moreover, they determine the intrinsic conformational flexibility which is typical of membrane interacting peptides. Moreover, membranotropic peptides present a high content of aromatic residues, whose side chains are able to form favourable interactions with phospholipid groups which are located at the membrane interface and thus contribute to the insertion of the peptide into the bilayer [33].

Their cellular uptake is strongly dependent on the secondary structure adopted after interaction with cellular plasma membrane. A key feature of these peptides is amphipathicity [34]. They can be amphipathic in their primary structure or secondary structure. While primary amphipathic peptides present a sequential assembly of hydrophobic and hydrophilic domains separated by a spacer, secondary amphipathic peptides present a secondary structure with hydrophobic and hydrophilic residues located on opposite sides. The two faces of secondary amphipathic peptides are often characterized by the presence of large and aromatic residues on one face with and small residues such as Ala/Gly on the other face. This distribution of amino acid residues enhances the interaction and insertion within the membrane bilayer [35]. Conformational polymorphism is also relevant for determining the uptake mechanism, in fact, the ability to change from random to α or β conformations following membrane interaction is a peculiar trait of this class of peptides [35].

Several structural models describing interactions between peptides and membranes have emerged in recent years, which include membrane permeabilization through the formation of stable pores (such as barrel-stave and toroidal pore models) or micellization on a detergent-like way (carpet model) [36]. The main differences between these models are the lipid structure around the pores and their stability. In the barrel-stave model, peptide helices form well-defined and stable bundles, which can serve as a pore. In the toroidal-pore model, the lipids create a pore covered with peptides in different orientations; as a consequence, these pores are less stable. In the carpet model, peptides accumulate, adsorb and span the bilayer surface until its integrity is violated and transient holes are formed allowing additional peptides to penetrate the membrane; as a consequence, the membrane disintegrates in a dispersive-like manner rather than channel formation, and peptides do not necessarily insert into the hydrophobic membrane core. Peptide-membrane interactions are complex and diverse phenomena and according to peptide composition, charge, and structure, different peptides may employ different interaction mechanisms with the membrane. Membranotropic CPPs’ strong affinity for the membrane bilayer is dominated by hydrophobic interactions. Thus, they penetrate deeper into the hydrophobic core compared to cationic CPPs but do not span the bilayer in a pore-like manner. On the contrary, they tend to self-associate at the interface between the membrane and the aqueous compartments which could be relevant for direct translocation. As a matter of fact, an inverted micelle model has been proposed for many membranotropic CPPs. This model cannot be applied for explaining the uptake of cationic CPPs which do not contain the hydrophobic amino acids necessary for the translocation process. In addition to the inverted micelles model, direct translocation can be achieved also via transient pore formation or carpet-like perturbations. Thus, direct translocation involves stable or transient destabilization of the membrane bilayer associated with folding of the peptide in the lipid membrane.

In general, the uptake mechanism may also depend on the CPP concentration. Cationic CPP uptake is achieved by endocytosis at low peptide concentrations but may switch to direct uptake above a certain threshold [37]; moreover, primary hydrophobic CPP direct penetration is more probable at high concentrations. On the contrary, membranotropic CPPs direct penetration likely takes place at both high and low CPP concentrations. Although the concentration threshold for direct penetration varies according to the different CPP, different cell lines, the presence and type of cargo and mainly the mechanism of interaction with the membrane bilayer.

**Figure 1:**
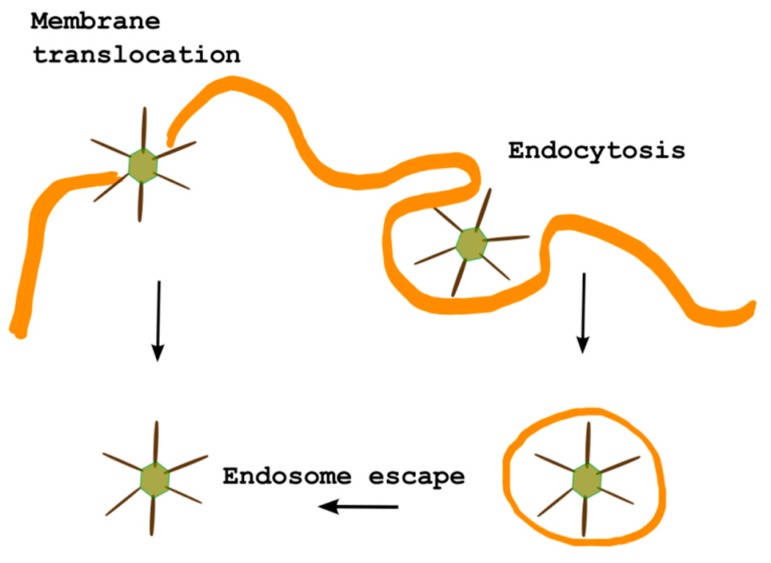
Mechanism of internalization of gH625 coupled to a cargo. In yellow are reported membrane bilayers, in green the cargo and in black the CPP.

It is widely accepted that the interaction of each CPP with cellular membranes determines their uptake efficiency, but when they carry a cargo inside cells, several factors have to be taken into consideration, spanning from the nature of the conjugated cargo (type, size, charge) to differences in the properties of the CPP (length, charge, hydrophobicity, secondary structure) as well as the cells lines under investigation and the concentrations of both the CPP and the cargo. Depending on these factors several internalization routes may act simultaneously. Nevertheless, cationic CPPs transport their cargo inside cells essentially using endocytosis. Thus, the cargo needs to escape from the endosomal vesicle in order to exert its action. If the cargo remains entrapped into the endosomes, it will end into lysosomes where degradation processes take place which abolish the biological effects of the cargo. On the contrary, membranotropic CPPs uptake of mechanisms mainly involve direct penetration of the plasma membrane and, consequently, immediate bioavailability of the cargo. In fact, hydrophobic peptides that partition into membranes are able to efficiently interact with membranes and enter cells which also includes the crossing of endothelial layers *in vivo* [21,38]. Moreover, these peptides are able to efficiently cross biological membranes, promote lipid-membrane reorganizing processes (fusion or pore formation) and determine a local and temporary membrane destabilization with subsequent reorganization [39,40]. Moreover, they can circumvent the endosomal entrapment both favouring the escape from the endosome and translocating a cargo through the plasma membrane directly into the cell (Figure 1). Modifying the internalization mechanism of a cargo will also modify the toxicity of the internalized drug and may be involved in the overcoming of drug resistance problems.

## 4. Examples of Membranotropic CPPs

CADY is a 20 residue peptide, which contains both aromatic tryptophan residues and cationic residues. It assumes an amphipathic helical conformation when in the bilayer, with charged residues on one side and Trp on the other. The helical conformation is involved in the interactions with the cell membrane and in the mechanism of cell penetration. CADY has been used to enhance cellular uptake of siRNA [31], and its internalization is barely affected by the use of inhibitors of the endosomal pathway.

MPG is a 27 amino acid peptide derived from the hydrophobic fusion domain of HIV gp41 which is thought to be responsible of cellular entry and the hydrophilic nuclear import sequence of SV40 T antigen which binds the cargo and also facilitates entry into the nucleus [28]. The glycine-rich HIV fusion peptide is essential for membrane fusion activity while the NLS of the SV40 large T antigen improves the nuclear targeting [41,42]. *In vitro* studies have shown that MPG can deliver both siRNA and DNA after just 1 h [43]. The hydrophobic domain is critical for insertion into membranes. MPG cellular uptake exploits different routes; nevertheless, the major cell translocation mechanism is independent of the endosomal pathway and temporarily involves membrane disorganization and folding into β-structures within the membrane bilayer without any associated leakage or toxicity; all these fenomena facilitate insertion into the membrane and initiation of translocation.

Pep-1 is partially derived from MPG; in fact, it conserves the C-terminal hydrophilic domain corresponding to the NLS of SV40 large T antigen; the hydrophobic region instead corresponds to a tryptophan-rich sequence (KETWWETWWTE) derived from the HIV-1 reverse transcriptase [22,23]. Pep-1 is able to efficiently deliver a variety of cargos into several cell lines in a fully active form. The strong interaction with the lipid bilayer, causes local perturbation and allows the peptide to cross the membrane by a physical mediated mechanism promoted by the transmembrane potential and not involving pore formation [22,44,45]. Pep-1 interaction with membranes is key for its cellular uptake and is correlated to a conformational change from random coil in water to α-helix in membrane. The hydrophobic domain is involved in this conformational change which as a consequence allows its easy insertion into the membrane. The peptide is able to transiently modify the cell-membrane organization without causing any leakage or toxicity, and thus smoothing insertion into the membrane and initiation of the translocation process. The ability to directly interact with lipids also limits the association with proteoglycans at the cell surface, accelerating cellular entry and reducing the extent of internalization through the endosomal pathway. This mechanism does not completely exclude the endocytic internalization but the length of time required for the translocation is considerably lower than entry by the endocytic pathway. Therefore, when both mechanisms are effective, the non-endocytic pathway is prevailing.

C105Y is a synthetic peptide based on the amino acid sequence of a1-antitrypsin and was shown to enter the cytoplasm, nucleus, and nucleolus of live cells very rapidly [26]. C105Y uptake and internalization does not occur through known endocytic pathways. C105Y attached to polyK was used to condense plasmid DNA and allowed a 100-fold increase in gene expression compared to polyK-DNA complexes that did not contain C105Y. This complex was also able to increase gene expression *in vivo*.

*Herpes Simplex Virus* Type 1 (HSV-1) Protein VP22 [46] is a major component of the tegument and has been reported to traffic between cells [47]; its C-terminal 40 amino acid domain has been shown to be responsible for the transduction property of the whole protein and its was demonstrated to be able to mediate the delivery of DNA and RNA oligonucleotides into cells [48,49].

The 4-kDa peptide called “gamma” (γ) derived from the insect *Flock House* virus (FHV) [50] contains an N terminal region that is able to alter membrane structure and increase bilayer permeability [51]. In fact, biophysical studies have shown that this domain presents membranotropic features and is able to strongly bind fluid phase lipid bilayers increasing transmembrane permeability. Its activity is similar to that of known membrane active peptides such as melittin and alamethicin [52]; thus, peptide-triggered disruption of the endosomal membrane may represent a premise to viral uncoating in the host cytoplasm. Comparisons with the TAT peptide showed that the FHV peptide was able to internalise with a higher efficiency [53].

Transportan and TP10 are synthetic peptides derived from the N-terminal domain of the neuropeptide galanin linked through a lysine residue to mastoparan. It translocates across the membrane bilayer and has been used for the transfer of several cargoes. The internalization is not correlated to the presence of a receptor, energy and temperature [27]. Moreover, it could involve both by endocytosis and direct translocation. Structural analysis shows that it adopts a helical conformation in the presence of the membrane bilayer.

pVEC is derived from the murine vascular endothelial-cadherin protein and has proved to be able to translocate into different cell lines. Direct translocation has been attributed to the presence of N-terminal hydrophobic residues [24].

INF [30] and GALA [32] have been shown to increase the transfection efficiencies; they are α-helical peptides with pH-dependent fusogenic and endosomolytic activities able to enhance lysosomal degradation before the contents of the endosomes are delivered to lysosomes.

VT5 is a synthetic water soluble amphipathic 26-mer β-sheet peptide which was shown to be able to enhance internalization into endothelial cells; the mechanism of entry of VT5 into the cells has not been revealed yet, but it was shown to be energy, temperature and pH dependent [25].

## 5. gH625, a Delivery Sequence Derived from Herpes Simplex Virus Type I

Among membranotropic peptides with a tendency to membrane binding and a high interfacial hydrophobicity or amphipathicity, those derived from enveloped virus glycoproteins are particularly challenging because they can physically interfere with the hydrophobic surfaces located on membranes and/or fusion proteins during the enveloped virus entry and are, thus, involved both in fusion and entry. Moreover, CPPs present in viruses that can infect many different hosts may have a wide applicability. The high content of aromatic residues is probably associated to the overcoming of the energy cost of peptide bond partitioning into membranes and determine the insertion of the peptide into just one leaflet of the bilayer [54]. This asymmetric insertion into only one membrane monolayer causes the formation of bulges that stick out of the membrane and ease contacts between fusing bilayers [55]. The entry of viruses involves several membrane reorganization processes such as transient permeabilization of the bilayer, which are similar to the ones involved in delivery across cellular membranes.

A milestone in understanding the role of hydrophobic viral peptides is figured out by the sequence “gH625” derived from glycoprotein H of *Herpes simplex virus* type I, developed in our laboratory [29]. HSV fusion proteins contain several membranotropic sequences with the ability to bind and disrupt model membranes [56,57,58,59,60,61,62,63,64,65,66], which are involved in the local destabilization of the membrane bilayer which determines the fusion of the viral and host cell membranes [58,60,66,67]. gH625 corresponds to the HSV-1 region with the highest fusion capability which was initially identified using the Wimley-White interfacial hydrophobicity scale (WWIH) and subsequent studies demonstrated the numerous use of this sequence including membrane fusion, viral inhibition and drug delivery [21,68,69].

gH625 is a membrane-perturbing domain, containing key residues for interacting and destabilizing biological membranes [64,66,70]. In fact, it is rich in hydrophobic residues including glycines, leucines, alanines, and aromatic residues such as tryptophan and tyrosines, which are known to be located preferentially at the membrane interface. The hydrophobic domain is fundamental for its insertion into the membrane and is involved in the early stages of membrane perturbation. The peptide gH625 is able to interact with model membranes, to insert into the bilayer from its N-terminal side; moreover, it has a tryptophan residue buried inside the bilayer, and adopts an amphiphatic helical conformation. The amphipathic helices would allow the peptide to enter the membrane, and trigger local fusion of the membrane leaflets, transient pore formation, cracks and membrane fusion [60,63,64,65].

The initial interactions with the membrane and the oligomerization process is favoured by the presence of the histidine residue at the N-terminus of the native sequence, which strongly increases the fusion activity [64]. In fact, the presence of any other residue at the N-terminus impaired the fusion ability of the peptide (data not published).

The hydrophobic and amphipathic profile of gH625 provides the necessary distinctive features for the interaction with membrane lipids and the formation of a transient helical structure that temporarily affects membrane organization, thereby facilitating insertion into the membrane and translocation [29,71]. gH625 has been shown to be able to directly translocate across the membrane bilayer and to transport into the cytosol several cargoes [21,29,38,39,71,72,73,74,75,76,77,78,79] (Figure 1).

gH625-QDs internalization was shown to be highly enhanced and to involve the endocytic pathway only to a minor extent [71]. Liposomes armed with gH625 on their external surface and loaded with doxorubicin (DOXO) [78] or mitoxantrone (MTX) [80], were shown to penetrate inside cells and influence the uptake mechanism of liposomes and the intracellular distribution and release of the drug. Moreover, the presence of gH625 was shown to determine an increased uptake also in DOXO resistant cells [81]. Poly(amide)-based dendrimers coupled to gH625 on the termini allowed the enhanced translocation of the conjugate [72,73,74]. A non-active translocation mechanism was shown to be involved in the internalization of peptide-functionalized dendrimers [73]. gH625 was also used to enhance the uptake of NPs by brain endothelial cells [38], and it decreased NP intracellular accumulation as large aggregates and enhanced the NP blood brain barrier crossing.

Recently, we evaluated the ability of gH625 to cross the BBB *in vivo* [79]. The peptide was administered *in vivo* to rats, and its presence in the liver and in the brain was analysed. Within 3.5 h from its i.v. administration, the peptide was found beyond the BBB in proximity of cell neurites. Moreover, it did not show toxic effects *in vivo*. In fact, it did not influence the brain maximal oxidative capacity and mitochondrial respiration rate. Our data further support the possible use of gH625 as a novel nano-carrier system for drug delivery to the central nervous system.

## 6. Conclusions

Synthetic peptides are particularly attractive candidate nanomaterials to be developed as delivery vectors since they have the flexibility to incorporate different functionalities. In fact, they can overcome extra and intracellular barriers, they can achieve tissue and cell type specificity, and they can be coupled covalently and non-covalently to a variety of cargoes and they generally show low cytotoxicity. Membranotropic CPPs represent a challenge for drug delivery as they can translocate directly across membranes. Direct translocation could be especially advantageous because they would be immediately available in the cytosol and the risk of endosomal entrapment and degradation would be eliminated. A further advantage in favour of membranotropic peptides is the fact that modifying the internalization mechanism and avoiding the endocytic pathway determines a reduction of toxicity and probably helps in overcoming drug resistance problems.

Despite many studies on CPPs, the mechanism by which different CPP enter cells has not been exactly determined. Unfortunately, there is no specific biological or biophysical method that could provide a comprehensive answer to all questions and, therefore, a combination of different methods and techniques is indispensable [76]. The *in vitro* and *in vivo* use of CPPs as drug delivery vectors is, however, limited by the lack of cell specificity; this means that a particular valuable goal in the medicinal field is the creation of a platform functionalized both with CPPs for enhancing uptake and targeting peptides for favouring specificity [82,83].

Although the number of studies devoted to CPPs is increasing, there is still space for improvements regarding the molecular mechanisms that underlie features such as their cellular uptake and membrane translocation, the efficacy in the presence of serum and enhancement of endosomal escape. Understanding all of these points is a necessary requirement to characterize the structural basis for modulation of these peptides and for all researchers involved in the design of novel theranostic systems.
